# Supplementary material for: Rural–Urban Inequalities in Poor Self-Rated Health, Self-Reported Functional Disabilities, and Depression among Chinese Older Adults: Evidence from the China Health and Retirement Longitudinal Study 2011 and 2015
Source: Int J Environ Res Public Health. 2021 Jun 18;18(12):6557. doi: 10.3390/ijerph18126557 (PMC8296324; doi:10.3390/ijerph18126557)
Supplement: Supplementary file 1 [file ijerph-18-06557-s001.zip › ijerph-1229507-supplementary.pdf]

# Supplementary tables

**Table S1.** Prevalence (%) of impaired Basic Activities of Daily Living (BADLs) among rural and urban respondents, CHARLS 2011 and CHARLS 2015.

| | **CHARLS 2011 (*n* = 5955)** | | | CHARLS 2015 (*n* = 7338) | | |
|---|---|---|---|---|---|---|
| | **Rural (*n* = 4337)** | **Urban (*n* = 1618)** | ***p*** | Rural (*n* = 5366) | Urban (*n* = 1972) | *p* |
| Total | 28.2 | 17.1 | <0.001 | 30.5 | 20.6 | <0.001 |
| Age group (years) | | | | | | |
| 60-64 | 21.5 | 10.2 | <0.001 | 22.6 | 13.6 | <0.001 |
| 65-69 | 25.6 | 16.0 | <0.001 | 29.1 | 16.3 | <0.001 |
| 70-74 | 31.9 | 16.7 | <0.001 | 34.7 | 21.5 | <0.001 |
| 75-79 | 36.7 | 19.2 | <0.001 | 39.5 | 29.3 | 0.004 |
| 80-84 | 45.5 | 41.2 | 0.457 | 45.1 | 33.3 | 0.025 |
| 85-89 | 49.4 | 57.6 | 0.425 | 48.9 | 56.3 | 0.381 |
| 90+ | 51.9 | 42.9 | 1.000 | 51.2 | 66.7 | 0.303 |
| Sex | | | | | | |
| Male | 24.0 | 16.1 | <0.001 | 25.5 | 19.1 | <0.001 |
| Female | 32.3 | 18.3 | <0.001 | 35.2 | 22.2 | <0.001 |
| Marital status | | | | | | |
| Married | 26.5 | 15.5 | <0.001 | 28.0 | 19.1 | <0.001 |
| Unmarried | 33.9 | 24.0 | 0.001 | 39.1 | 27.4 | 0.001 |
| Living arrangement | | | | | | |
| Not living alone | 27.8 | 16.9 | <0.001 | 30.0 | 20.3 | <0.001 |
| Living alone | 31.4 | 18.5 | 0.001 | 35.2 | 24.5 | 0.016 |
| Living near children | | | | | | |
| Living near children | 27.9 | 17.6 | <0.001 | 30.9 | 21.4 | <0.001 |
| Not living near children | 31.8 | 6.5 | <0.001 | 26.3 | 14.6 | 0.001 |
| No child | 26.4 | 18.0 | 0.261 | 34.3 | 13.8 | 0.033 |
| Educational level | | | | | | |
| Below primary school | 31.6 | 25.1 | 0.006 | 33.6 | 29.9 | 0.119 |
| Primary school | 22.7 | 16.8 | 0.016 | 27.3 | 22.5 | 0.033 |
| Middle school | 19.0 | 13.7 | 0.050 | 21.9 | 15.6 | 0.009 |
| High school | 10.8 | 10.7 | 0.995 | 13.3 | 17.8 | 0.242 |
| College and above | 0.0 | 15.8 | 1.000 | 0.0 | 9.9 | 1.000 |
| Income group | | | | | | |
| First group (low) | 33.7 | 28.8 | 0.411 | 31.4 | 19.8 | 0.017 |
| Second group | 33.8 | 36.1 | 0.770 | 28.3 | 38.5 | 0.262 |
| Third group | 25.1 | 27.0 | 0.686 | 28.6 | 28.9 | 0.965 |
| Fourth group | 20.2 | 20.0 | 0.944 | 25.7 | 21.3 | 0.284 |
| Fifth group (high) | 14.9 | 12.0 | 0.209 | 19.9 | 16.5 | 0.273 |
| Missing | 28.8 | 20.4 | 0.007 | 33.0 | 21.6 | <0.001 |

**Table S2.** Prevalence (%) of impaired Instrumental Activities of Daily Living (IADLs) among rural and urban respondents, CHARLS 2011 and CHARLS 2015.

| | **CHARLS 2011 (*n* = 5963)** | | | CHARLS 2015 (*n* = 7192) | | |
|---|---|---|---|---|---|---|
| | **Rural (*n* = 4336)** | **Urban (*n* = 1627)** | ***p*** | Rural (*n* = 5237) | Urban (*n* = 1955) | *p* |
| Total | 33.9 | 21.3 | <0.001 | 33.6 | 20.8 | <0.001 |
| Age group (years) | | | | | | |
| 60-64 | 24.5 | 13.3 | <0.001 | 25.4 | 12.4 | <0.001 |
| 65-69 | 30.5 | 18.5 | <0.001 | 31.4 | 17.3 | <0.001 |
| 70-74 | 38.7 | 20.0 | <0.001 | 36.2 | 22.6 | <0.001 |
| 75-79 | 45.5 | 26.4 | <0.001 | 44.4 | 29.0 | <0.001 |
| 80-84 | 57.6 | 52.0 | 0.341 | 54.8 | 34.7 | <0.001 |
| 85-89 | 69.8 | 69.7 | 0.994 | 59.5 | 60.9 | 0.873 |
| 90+ | 80.8 | 57.1 | 1.000 | 56.1 | 80.0 | 0.102 |
| Sex | | | | | | |
| Male | 27.2 | 19.0 | <0.001 | 26.5 | 17.8 | <0.001 |
| Female | 40.6 | 23.9 | <0.001 | 40.3 | 24.1 | <0.001 |
| Marital status | | | | | | |
| Married | 30.6 | 19.8 | <0.001 | 30.7 | 19.4 | <0.001 |
| Unmarried | 45.0 | 28.1 | <0.001 | 43.9 | 27.4 | <0.001 |
| Living arrangement | | | | | | |
| Not living alone | 34.0 | 21.4 | <0.001 | 33.4 | 20.6 | <0.001 |
| Living alone | 33.3 | 20.9 | 0.001 | 35.7 | 23.2 | 0.006 |
| Living near children | | | | | | |
| Living near children | 34.1 | 21.4 | <0.001 | 34.4 | 21.8 | <0.001 |
| Not living near children | 31.2 | 14.1 | 0.003 | 27.2 | 11.6 | <0.001 |
| No child | 35.2 | 31.4 | 0.647 | 35.3 | 27.6 | 0.438 |
| Educational level | | | | | | |
| Below primary school | 39.1 | 33.3 | 0.021 | 38.4 | 32.4 | 0.015 |
| Primary school | 24.9 | 21.9 | 0.228 | 27.1 | 22.5 | 0.042 |
| Middle school | 20.3 | 16.3 | 0.163 | 21.3 | 15.8 | 0.023 |
| High school | 13.8 | 14.0 | 0.971 | 18.9 | 15.3 | 0.355 |
| College and above | 0.0 | 11.2 | 1.000 | 16.7 | 10.7 | 0.648 |
| Income group | | | | | | |
| First group (low) | 38.3 | 36.4 | 0.752 | 31.2 | 18.1 | 0.007 |
| Second group | 39.5 | 44.4 | 0.549 | 29.4 | 36.0 | 0.481 |
| Third group | 30.5 | 31.7 | 0.807 | 30.2 | 44.7 | 0.060 |
| Fourth group | 28.0 | 25.3 | 0.389 | 28.9 | 15.7 | 0.002 |
| Fifth group (high) | 23.4 | 17.1 | 0.021 | 18.2 | 13.5 | 0.106 |
| Missing | 33.8 | 19.1 | <0.001 | 38.1 | 23.7 | <0.001 |

**Table S3.** Prevalence (%) of self-reported depression among rural and urban respondents, CHARLS 2011 and CHARLS 2015

| | **CHARLS 2011 (*n* = 5567)** | | | CHARLS 2015 (*n* = 6811) | | |
|---|---|---|---|---|---|---|
| | **Rural (*n* = 4004)** | **Urban (*n* = 1523)** | *p* | Rural (*n* = 4999) | Urban (*n* = 1812) | *p* |
| Total | 47.4 | 27.8 | <0.001 | 40.5 | 22.7 | <0.001 |
| Age group (years) | | | | | | |
| 60-64 | 45.6 | 27.4 | <0.001 | 38.4 | 21.4 | <0.001 |
| 65-69 | 47.4 | 29.2 | <0.001 | 43.9 | 22.6 | <0.001 |
| 70-74 | 48.9 | 25.9 | <0.001 | 42.0 | 25.6 | <0.001 |
| 75-79 | 48.0 | 23.0 | <0.001 | 39.7 | 24.2 | <0.001 |
| 80-84 | 51.2 | 37.8 | 0.039 | 36.6 | 20.0 | 0.003 |
| 85-89 | 55.1 | 43.5 | 0.335 | 36.1 | 15.6 | 0.032 |
| 90+ | 47.1 | 42.9 | 1.000 | 33.3 | 40.0 | 1.000 |
| Sex | | | | | | |
| Male | 39.5 | 20.5 | <0.001 | 32.4 | 19.1 | <0.001 |
| Female | 55.0 | 35.6 | <0.001 | 48.4 | 26.6 | <0.001 |
| Marital status | | | | | | |
| Married | 45.1 | 25.3 | <0.001 | 38.3 | 21.4 | <0.001 |
| Unmarried | 55.3 | 38.5 | <0.001 | 49.1 | 28.7 | <0.001 |
| Living arrangement | | | | | | |
| Not living alone | 46.8 | 26.6 | <0.001 | 40.0 | 22.4 | <0.001 |
| Living alone | 52.9 | 35.5 | <0.001 | 46.3 | 26.3 | <0.001 |
| Living near children | | | | | | |
| Living near children | 46.6 | 27.7 | <0.001 | 40.0 | 22.6 | <0.001 |
| Not living near children | 57.4 | 25.0 | <0.001 | 43.3 | 22.5 | <0.001 |
| No child | 46.5 | 34.0 | 0.164 | 48.4 | 34.8 | 0.241 |
| Educational level | | | | | | |
| Below primary school | 51.2 | 37.5 | <0.001 | 44.8 | 31.8 | <0.001 |
| Primary school | 41.5 | 31.9 | 0.002 | 35.3 | 24.1 | <0.001 |
| Middle school | 35.7 | 24.5 | 0.001 | 29.6 | 19.5 | <0.001 |
| High school | 31.7 | 18.4 | 0.022 | 26.7 | 17.9 | 0.041 |
| College and above | 25.0 | 14.7 | 0.483 | 0.0 | 14.8 | 0.308 |
| Income group | | | | | | |
| First group (low) | 50.8 | 49.2 | 0.806 | 44.8 | 23.6 | <0.001 |
| Second group | 53.5 | 41.2 | 0.157 | 41.9 | 23.1 | 0.056 |
| Third group | 45.7 | 44.7 | 0.845 | 43.2 | 39.5 | 0.651 |
| Fourth group | 42.4 | 29.3 | <0.001 | 36.2 | 32.6 | 0.430 |
| Fifth group (high) | 32.7 | 22.9 | 0.002 | 30.9 | 16.7 | <0.001 |
| Missing | 46.1 | 27.7 | <0.001 | 40.4 | 23.6 | <0.001 |

**Table S4.** Multiple logistic regression analyses on rural and urban respondents' impaired Basic Activities of Daily Living (BADLs), adjusted for age and sex, CHARLS 2011 ($n$ = 5995)

| | **Model 1** | | **Model 2** | | **Model 3** | | **Model 4** | | **Model 5** | |
|---|---|---|---|---|---|---|---|---|---|---|
| | OR | 95% CI | OR | 95% CI | OR | 95% CI | OR | 95% CI | OR | 95% CI |
| Rural respondents [a] | 1.99 | 1.72-2.31 | 1.96 | 1.69-2.28 | 1.59 | 1.34-1.89 | 1.24 | 1.03-1.48 | 1.09 | 0.90-1.32 |
| Marital status [b] | | | | | | | | | | |
| Unmarried | — | — | 1.08 | 0.91-1.29 | 1.07 | 0.90-1.27 | 1.10 | 0.93-1.32 | 1.09 | 0.91-1.30 |
| Living arrangement [c] | | | | | | | | | | |
| Living alone | — | — | 0.82 | 0.65-1.04 | 0.82 | 0.65-1.04 | 0.72 | 0.57-0.92 | 0.73 | 0.57-0.92 |
| Living near children [d] | | | | | | | | | | |
| Not living near children | — | — | 1.25 | 0.98-1.59 | 1.25 | 0.98-1.60 | 1.19 | 0.93-1.52 | 1.19 | 0.93-1.52 |
| No child | — | — | 1.07 | 0.71-1.62 | 1.05 | 0.69-1.58 | 1.07 | 0.71-1.62 | 1.05 | 0.69-1.60 |
| Educational level [e] | | | | | | | | | | |
| Primary school | — | — | — | — | 0.77 | 0.65-0.90 | — | — | 0.79 | 0.67-0.93 |
| Middle school | — | — | — | — | 0.66 | 0.53-0.84 | — | — | 0.73 | 0.58-0.91 |
| High school | — | — | — | — | 0.44 | 0.30-0.64 | — | — | 0.52 | 0.35-0.76 |
| College and above | — | — | — | — | 0.66 | 0.40-1.09 | — | — | 0.81 | 0.49-1.35 |
| Income group [f] | | | | | | | | | | |
| Second group | — | — | — | — | — | — | 1.09 | 0.90-1.32 | 1.08 | 0.90-1.31 |
| Third group | — | — | — | — | — | — | 0.76 | 0.62-0.93 | 0.76 | 0.62-0.93 |
| Fourth group | — | — | — | — | — | — | 0.59 | 0.47-0.73 | 0.59 | 0.48-0.74 |
| Fifth group (high) | — | — | — | — | — | — | 0.35 | 0.27-0.46 | 0.38 | 0.29-0.49 |

| | | | | | | | | | | |
|---|---|---|---|---|---|---|---|---|---|---|
| Missing | — | — | — | — | — | — | 0.80 | 0.65-0.99 | 0.81 | 0.66-0.99 |

OR: odds ratio; CI: confidence interval 95%; [a] Reference group: urban respondents; [b] Reference group= unmarried; [c] Reference group= not living alone; [d] Reference group= living near children; [e] Reference group= below primary school; [f] Reference group= first group (low)

**Table S5.** Multiple logistic regression analyses on rural and urban respondents' impaired Basic Activities of Daily Living (BADLs), adjusted for age and sex, CHARLS 2015 ($n$ = 7338)

| | **Model 1** | | **Model 2** | | **Model 3** | |
|---|---|---|---|---|---|---|
| | OR | 95% CI | OR | 95% CI | OR | 95% CI |
| Rural respondents [a] | 1.76 | 1.55-2.00 | 1.74 | 1.54-1.98 | 1.43 | 1.24-1.65 |
| Marital status [b] | | | | | | |
| Unmarried | — | — | 1.22 | 1.05-1.42 | 1.20 | 1.03-1.40 |
| Living arrangement [c] | | | | | | |
| Living alone | — | — | 0.80 | 0.64-0.99 | 0.80 | 0.64-0.99 |
| Living near children [d] | | | | | | |
| Not living near children | — | — | 0.96 | 0.80-1.15 | 0.97 | 0.81-1.17 |
| No child | — | — | 1.24 | 0.83-1.83 | 1.19 | 0.80-1.76 |
| Educational level [e] | | | | | | |
| Primary school | — | — | — | — | 0.86 | 0.75-0.98 |
| Middle school | — | — | — | — | 0.68 | 0.57-0.82 |
| High school | — | — | — | — | 0.61 | 0.47-0.81 |
| College and above | — | — | — | — | 0.31 | 0.18-0.55 |

OR: odds ratio; CI: confidence interval 95%;

[a] Reference group: urban respondents; [b] Reference group= unmarried; [c] Reference group= not living alone; [d] Reference group= living near children; [e] Reference group= below primary school

**Table S6.** Multiple logistic regression analyses on rural and urban respondents' impaired Instrumental Activities of Daily Living (IADLs), adjusted for age and sex, CHARLS 2011 ($n$ = 5963)

| | **Model 1** | | **Model 2** | | **Model 3** | | **Model 4** | | **Model 5** | |
|---|---|---|---|---|---|---|---|---|---|---|
| | OR | 95% CI | OR | 95% CI | OR | 95% CI | OR | 95% CI | OR | 95% CI |
| Rural respondents [a] | 2.05 | 1.78-2.35 | 1.97 | 1.71-2.27 | 1.46 | 1.25-1.72 | 1.40 | 1.19-1.66 | 1.13 | 0.95-1.36 |
| Marital status [b] | | | | | | | | | | |
| Unmarried | — | — | 1.44 | 1.22-1.70 | 1.41 | 1.19-1.67 | 1.45 | 1.23-1.73 | 1.43 | 1.21-1.70 |
| Living arrangement [c] | | | | | | | | | | |
| Living alone | — | — | 0.48 | 0.38-0.60 | 0.47 | 0.37-0.59 | 0.43 | 0.34-0.55 | 0.43 | 0.34-0.55 |
| Living near children [d] | | | | | | | | | | |
| Not living near children | — | — | 1.07 | 0.84-1.36 | 1.08 | 0.85-1.38 | 1.02 | 0.80-1.30 | 1.03 | 0.80-1.31 |
| No child | — | — | 1.59 | 1.09-2.33 | 1.57 | 1.07-2.30 | 1.59 | 1.08-2.33 | 1.57 | 1.07-2.31 |
| Educational level [e] | | | | | | | | | | |
| Primary school | — | — | — | — | 0.68 | 0.59-0.80 | — | — | 0.70 | 0.60-0.81 |
| Middle school | — | — | — | — | 0.57 | 0.46-0.71 | — | — | 0.60 | 0.48-0.75 |
| High school | — | — | — | — | 0.42 | 0.30-0.59 | — | — | 0.47 | 0.33-0.67 |
| College and above | — | — | — | — | 0.31 | 0.17-0.54 | — | — | 0.35 | 0.20-0.62 |
| Income group [f] | | | | | | | | | | |
| Second group | — | — | — | — | — | — | 1.18 | 0.98-1.43 | 1.17 | 0.97-1.41 |
| Third group | — | — | — | — | — | — | 0.81 | 0.66-0.99 | 0.81 | 0.66-0.98 |
| Fourth group | — | — | — | — | — | — | 0.73 | 0.59-0.90 | 0.73 | 0.59-0.90 |
| Fifth group (high) | — | — | — | — | — | — | 0.50 | 0.39-0.63 | 0.55 | 0.43-0.70 |

| | | | | | | | | | | |
|---|---|---|---|---|---|---|---|---|---|---|
| Missing | — | — | — | — | — | — | 0.79 | 0.64-0.96 | 0.80 | 0.65-0.98 |

OR: odds ratio; CI: confidence interval 95%;

[a] Reference group: urban respondents; [b] Reference group= unmarried; [c] Reference group= not living alone; [d] Reference group= living near children; [e] Reference group= below primary school; [f] Reference group= first group (low)

**Table S7.** Multiple logistic regression analyses on rural and urban respondents' impaired Instrumental Activities of Daily Living (IADLs), adjusted for age and sex, CHARLS 2015 ($n$ = 7192)

| | **Model 1** | | **Model 2** | | **Model 3** | |
|---|---|---|---|---|---|---|
| | OR | 95% CI | OR | 95% CI | OR | 95% CI |
| Rural respondents [a] | 2.03 | 1.79-2.31 | 2.02 | 1.78-2.30 | 1.59 | 1.38-1.84 |
| Marital status [b] | | | | | | |
| Unmarried | — | — | 1.27 | 1.09-1.49 | 1.25 | 1.07-1.45 |
| Living arrangement [c] | | | | | | |
| Living alone | — | — | 0.62 | 0.50-0.78 | 0.62 | 0.50-0.78 |
| Living near children [d] | | | | | | |
| Not living near children | — | — | 0.86 | 0.72-1.04 | 0.87 | 0.72-1.05 |
| No child | — | — | 1.46 | 0.99-2.15 | 1.38 | 0.93-2.04 |
| Educational level [e] | | | | | | |
| Primary school | — | — | — | — | 0.75 | 0.65-0.86 |
| Middle school | — | — | — | — | 0.61 | 0.51-0.74 |
| High school | — | — | — | — | 0.56 | 0.42-0.74 |
| College and above | — | — | — | — | 0.34 | 0.20-0.59 |

OR: odds ratio; CI: confidence interval 95%;

[a] Reference group: urban respondents; [b] Reference group= unmarried; [c] Reference group= not living alone; [d] Reference group= living near children; [e] Reference group= below primary school

**Table S8.** Multiple logistic regression analyses on rural and urban respondents' self-reported depression, adjusted for age and sex, CHARLS 2011 ($n$ = 5567)

| | **Model 1** | | **Model 2** | | **Model 3** | | **Model 4** | | **Model 5** | |
|---|---|---|---|---|---|---|---|---|---|---|
| | OR | 95% CI | OR | 95% CI | OR | 95% CI | OR | 95% CI | OR | 95% CI |
| Rural respondents [a] | 2.36 | 2.07-2.69 | 2.32 | 2.03-2.64 | 1.81 | 1.56-2.10 | 1.63 | 1.40-1.91 | 1.38 | 1.17-1.63 |
| Marital status [b] | | | | | | | | | | |
| Unmarried | — | — | 1.39 | 1.18-1.65 | 1.37 | 1.16-1.62 | 1.40 | 1.19-1.66 | 1.39 | 1.17-1.64 |
| Living arrangement [c] | | | | | | | | | | |
| Living alone | — | — | 0.97 | 0.78-1.21 | 0.97 | 0.78-1.20 | 0.90 | 0.72-1.13 | 0.91 | 0.73-1.13 |
| Living near children [d] | | | | | | | | | | |
| Not living near children | — | — | 1.55 | 1.24-1.93 | 1.55 | 1.24-1.94 | 1.48 | 1.18-1.85 | 1.49 | 1.18-1.86 |
| No child | — | — | 1.12 | 0.78-1.62 | 1.10 | 0.76-1.59 | 1.11 | 0.76-1.61 | 1.09 | 0.75-1.58 |
| Educational level [e] | | | | | | | | | | |
| Primary school | — | — | — | — | 0.84 | 0.73-0.96 | — | — | 0.86 | 0.74-0.99 |
| Middle school | — | — | — | — | 0.65 | 0.53-0.79 | — | — | 0.69 | 0.56-0.84 |
| High school | — | — | — | — | 0.49 | 0.36-0.66 | — | — | 0.55 | 0.40-0.75 |
| College and above | — | — | — | — | 0.37 | 0.22-0.61 | — | — | 0.43 | 0.26-0.72 |
| Income group [f] | | | | | | | | | | |
| Second group | — | — | — | — | — | — | 1.12 | 0.93-1.35 | 1.12 | 0.93-1.34 |
| Third group | — | — | — | — | — | — | 0.86 | 0.71-1.04 | 0.86 | 0.71-1.04 |
| Fourth group | — | — | — | — | — | — | 0.70 | 0.58-0.86 | 0.71 | 0.58-0.87 |
| Fifth group (high) | — | — | — | — | — | — | 0.47 | 0.38-0.59 | 0.52 | 0.41-0.65 |

| | | | | | | | | | | |
|---|---|---|---|---|---|---|---|---|---|---|
| Missing | — | — | — | — | — | — | 0.77 | 0.63-0.94 | 0.79 | 0.65-0.96 |

OR: odds ratio; CI: confidence interval 95%;

[a] Reference group: urban respondents; [b] Reference group= unmarried; [c] Reference group= not living alone; [d] Reference group= living near children; [e] Reference group= below primary school; [f] Reference group= first group (low)

**Table S9.** Multiple logistic regression analyses on rural and urban respondents' self-reported depression, adjusted for age and sex, CHARLS 2015 ($n$ = 6811)

| | **Model 1** | | **Model 2** | | **Model 3** | |
|---|---|---|---|---|---|---|
| | OR | 95% CI | OR | 95% CI | OR | 95% CI |
| Rural respondents [a] | 2.32 | 2.05-2.63 | 2.29 | 2.02-2.60 | 1.84 | 1.60-2.12 |
| Marital status [b] | | | | | | |
| Unmarried | — | — | 1.51 | 1.29-1.76 | 1.48 | 1.26-1.73 |
| Living arrangement [c] | | | | | | |
| Living alone | — | — | 0.85 | 0.68-1.05 | 0.85 | 0.68-1.05 |
| Living near children [d] | | | | | | |
| Not living near children | — | — | 1.22 | 1.04-1.45 | 1.24 | 1.05-1.47 |
| No child | — | — | 1.62 | 1.10-2.38 | 1.54 | 1.05-2.27 |
| Educational level [e] | | | | | | |
| Primary school | — | — | — | — | 0.77 | 0.68-0.88 |
| Middle school | — | — | — | — | 0.62 | 0.52-0.74 |
| High school | — | — | — | — | 0.57 | 0.43-0.74 |
| College and above | — | — | — | — | 0.44 | 0.26-0.73 |

OR: odds ratio; CI: confidence interval 95%;

[a] Reference group: urban respondents; [b] Reference group= unmarried; [c] Reference group= not living alone; [d] Reference group= living near children; [e] Reference group= below primary school
